# Benighted

**Published:** 1888-09-22

**Authors:** Lina Forster


### BENIGHTED.

"Ye are my disciples if ye love one another."

"I CANNOT come and let thee in," I cried.
"Within some other home thou must abide,
"There satisfy thy soul."
Again I heard the knocking—yet again—
And then there came the sound of rushing rain—
And the wild thunder's roll.

"I cannot come, and let thee in," I cried.
"Woe! woe is me! my little child hath died,
"And joy hath fled my life."
Again the knocking—then a cry of pain—
Once more I heard the sound of rushing rain,
And the fierce winds at strife.

At length I rose, and opened wide my door.
There I beheld, all sorrowful and poor—
Jesus, with wounded breast!
Then did I spread mine hands, and draw him in.
Low bowing down, I sore bewailed my sin,
Till Mercy gave me rest. —*Lina Forster.*

